# Draft Genome Sequence of Sicyoidochytrium minutum DNA Virus Strain 001

**DOI:** 10.1128/MRA.00418-21

**Published:** 2021-06-10

**Authors:** Yumi Murakoshi, Takayuki Shimeki, Daiske Honda, Yoshitake Takao

**Affiliations:** a Department of Marine Science and Technology, Fukui Prefectural University, Obama, Fukui, Japan; b Department of Biology, Faculty of Science and Engineering, Konan University, Kobe, Hyogo, Japan; c Institute for Integrative Neurobiology, Konan University, Kobe, Hyogo, Japan; Queens College CUNY

## Abstract

Sicyoidochytrium minutum DNA virus strain 001 (SmDNAV 001) is a double-stranded DNA (dsDNA) virus that infects the marine fungoid protist Sicyoidochytrium minutum. We report the draft genome sequence of SmDNAV 001. The 236,345-bp genome contained 358 coding sequences (CDSs) and 3 tRNA-coding sequences.

## ANNOUNCEMENT

Sicyoidochytrium minutum is a nonphotosynthetic stramenopile protist that belongs to the class Labyrinthulomycetes, family Thraustochytriaceae (1). Thraustochytrids are cosmopolitan osmotrophic or heterotrophic microorganisms that play essential roles in organic matter decomposition in marine environments (2). *Sicyoidochytrium minutum* DNA virus (SmDNAV) strain 001 was isolated from the estuary of the Shukugawa River (34.72°N, 135.32°E), Japan, in July 2003 (3). Its genome size was previously estimated as approximately 200 kbp, similar to that of other double-stranded DNA (dsDNA) viruses infecting microalgae (the family *Phycodnaviridae*) (4, 5). However, the morphology of SmDNAV differs considerably from that of other nucleocytoplasmic large DNA viruses (NCLDVs). The virion has a squashed ball-like shape and can change its morphology intra- and extracellularly (3).

Here, we report the draft genome sequence of SmDNAV strain 001. Genome DNA preparation was performed according to the method of Takao et al. (3). *S. minutum* NBRC 102975 and SmDNAV 001 were cultivated in 10× medium-H at 20°C. Virions were purified using the polyethylene glycol (PEG) concentration and ultracentrifugation method. The purified virions were treated with proteinase K and the DNA was extracted by the phenol/chloroform/isoamyl alcohol (P/C/I) method. After that, the DNA solution was treated with cetyltrimethylammonium bromide and extracted again by the P/C/I method. Genome DNA was fragmented using a Covaris system with its 500-bp fragmentation program. A DNA library was prepared using a KAPA Hyper Plus kit (Kapa Biosystems) and FastGene adapter kit (Nippon Genetics) according to the manufacturer’s instructions, and the library’s quality was confirmed using a fragment analyzer and the dsDNA 915 reagent kit (Advanced Analytical Technologies). A total of 277,712 paired-end reads were obtained from sequencing using a MiSeq instrument in 300-bp paired-end mode. Sickle version 1.33 (6) was used to remove bases with a quality value of less than 20; reads shorter than 127 bases and their paired reads were discarded, yielding 247,963 high-quality paired-end reads. A single linear contig was generated from *de novo* assembly using Unicycler version 0.3.0 (7). The size of 236,345 bp (average coverage depth, 398.151×) coincided with that of the SmDNAV 001 genome (ca. 200 kbp) previously estimated (3). Default parameters were used for all software unless otherwise specified.

The draft genome sequence of SmDNAV 001 was 236,345 bp long, and the GC content was 50.3%. Three tRNAs and 358 coding sequences (CDSs) were predicted from the SmDNAV 001 genome using tRNAscan-SE (8) and Glimmer version 0.3.2 (9). A large proportion of the CDSs were not similar to proteins in the public databases. Only 15% of the CDSs displayed translated amino acid-protein alignments that matched with an E value lower than 1e−5 using the BLASTX basic local alignment search tool against the NCBI nonredundant protein database. A total of 31 CDSs had hits to viral genes, with hits to NCLDVs for 28 CDSs. Seventeen and nineteen CDSs had hits to *Mimiviridae* and *Phycodnaviridae*, respectively. Only a single CDS displayed a hit to a *Phycodnaviridae* major capsid protein (GenBank accession number YP_009507580.1; E value, 2.78e−28) and *Mimivirus* major capsid protein (QBK88991.1; E value, 1.26e−20). It was notable that no homologue was identified for DNA polymerase, a common core gene in the NCLDV genome.

### Data availability.

The whole-genome sequencing data are available through the DDBJ Sequence Read Archive (DRA accession number DRA011919, BioProject number PRJDB11564, and BioSample number SAMD00317639), and the annotated genome assembly is also available (accession number LC627114, available from DDBJ).
